# Preferential targeting of MCL-1 by a hydrocarbon-stapled BIM BH3 peptide

**DOI:** 10.18632/oncotarget.27262

**Published:** 2019-10-22

**Authors:** Abbas Hadji, Greta K. Schmitt, Mathew R. Schnorenberg, Lauren Roach, Connie M. Hickey, Logan B. Leak, Matthew V. Tirrell, James L. LaBelle

**Affiliations:** ^1^Department of Pediatrics, Section of Hematology/Oncology/Stem Cell Transplantation and Committee on Cancer Biology, University of Chicago, Chicago, IL 60637, USA; ^2^Pritzker School of Molecular Engineering, University of Chicago, Chicago, IL 60637, USA

**Keywords:** MCL-1, BIM, BH3 mimetic, stapled peptides, apoptosis

## Abstract

BCL-2 family proteins are central regulators of apoptosis and represent prime therapeutic targets for overcoming cell death resistance in malignancies. However, plasticity of anti-apoptotic members, such as MCL-1, often allows for a switch in cell death dependency patterns that lie outside the binding profile of targeted BH3-mimetics. Therefore discovery of therapeutics that effectively inactivate all anti-apoptotic members is a high priority. To address this we tested the potency of a hydrocarbon stapled BIM BH3 peptide (BIM SAHB_*A*_) to overcome both BCL-2 and MCL-1 apoptotic resistance given BIM’s naturally wide ranging affinity for all BCL-2 family multidomain members. BIM SAHB_*A*_ effectively killed diffuse large B-cell lymphoma (DLBCL) cell lines regardless of their anti-apoptotic dependence. Despite BIM BH3’s ability to bind all BCL-2 anti-apoptotic proteins, BIM SAHB_*A*_’s dominant intracellular target was MCL-1 and this specificity was exploited in sequenced combination BH3-mimetic treatments targeting BCL-2, BCL-X_L_, and BCL-W. Extending this MCL-1 functional dependence, mouse embryonic fibroblasts (MEFs) deficient in MCL-1 were resistant to mitochondrial changes induced by BIM SAHB_*A*_. This study demonstrates the importance of understanding BH3 mimetic functional intracellular affinities for optimized use and highlights the diagnostic and therapeutic promise of a BIM BH3 peptide mimetic as a potential MCL-1 inhibitor.

## INTRODUCTION

The BCL-2 family can be divided into multidomain anti-apoptotic (*e.g.* BCL-2, BCL-X_L_, BCL-W, MCL-1, BFL-1) and pro-apoptotic (*e.g.* BAK, BAX) proteins. The functional interactions between these anti- and pro-apoptotic partners is controlled by a third group of proteins known as BH3-only proteins (*e.g.* BIM, BID, PUMA, BIK, BAD, NOXA, BMF) which contain one of four conserved BCL-2 homology (BH) domains. BH3-only proteins can directly bind and activate BAX/BAK or can insert their amphipathic BH3 α-helix into a groove on anti-apoptotic protein target(s) resulting in release and subsequent indirect BAX/BAK activation [1]. Cancer cells have long been known to evade cell death through overexpression of anti-apoptotic BCL-2 members or through down-regulation of BH3-only proteins [1]. To overcome these hurdles there is a great pharmacologic crusade to develop agents that directly engage BCL-2 family proteins to induce death regardless of the cell’s origin or genetic perturbations [2]. Despite early promise, many BH3-mimetics, have not effectively translated to the clinic or have been proven to work, at least in part, independent of the BCL-2 network [3–5].

Functional redundancy within the BCL-2 family can make it challenging to tailor effective therapeutic strategies without incurring resistance through upregulation of BCL-2 proteins that lie outside the mimetic’s binding profile [3, 6–9]. This is exemplified by diffuse large B-cell lymphoma (DLBCL) where MCL-1 contributes to intrinsic and acquired resistance to the rationally designed polyselective BCL-2, BCL-X_L_, and BCL-W inhibitor ABT-737 and the monoselective BCL-2 inhibitor ABT-199 [10, 11]. Despite the predominance of BCL-2 protein expression in DLBCL, either through the t(14;18) translocation and/or elevated *BCL2* copy numbers, many BCL-2^*High*^ DLBCL are resistant to direct BCL-2 inhibition and ultimately rely on MCL-1 for survival [11]. Additionally, although activated B-cell-like (ABC) DLBCL may rely on MCL-1 to a greater extent than germinal center B-cell-like (GCB) DLBCL, protein expression alone fails to predict reliance on BCL-2 or MCL-1 in either subtype. Rather, functional sequestration of pro-apoptotic BAK and BIM appear to define sensitivity to BH3-mimetic treatment [10, 12]. The importance of releasing BIM for cell death activation is exemplified by the treatment of BCL-2^*High*^ DLBCL with ABT-199 or the BCL-X_L_-selective inhibitor A-1155463 which results in ejection of BIM from these proteins but subsequent sequestration by MCL-1 [11]. The significance of this paradigm is reflected in encouraging results using BCL-2/BCL-X_L_ targeting BH3-mimetics in combination with agents that down-regulate MCL-1 in murine models of *MYC-BCL2* double-hit lymphoma and human DLBCL [13, 14]. It is clear that release of endogenous BIM sequestered by multiple anti-apoptotics is key to overcoming cell death resistance in diseases such as DLBCL.

The physiologic dominance of BIM in regulating apoptosis in hematopoietic cells is reflected in the ability of its BH3 death domain to tightly engage the BH3-binding groove of all anti-apoptotic proteins and directly activate BAX and BAK [15]. To exploit BIM’s natural death-inducing functions we and others have shown that a hydrocarbon-stapled peptide modeled after the BIM BH3 α-helix (BIM SAHB_*A*_) broadly targets BCL-2 proteins with high affinity *ex vivo*, blocks inhibitory anti-apoptotic interactions, directly triggers BAX activation, dissociates BAK from MCL-1, and induces dose-responsive and BH3 sequence–specific cell death in hematologic cancers [16–18]. In the present study, we investigated the effect of BIM SAHB_*A*_ on human DLBCL that differentially express and functionally depend on various BCL-2 anti-apoptotic proteins for survival [10]. We found that BIM SAHB_*A*_ induced apoptosis in DLBCL regardless of anti-apoptotic protein expression but that it did so most effectively in DLBCL that were increasingly resistant to ABT-737 and ABT-199. These results led to the finding that BIM SAHB_*A*_ preferentially displaced endogenous BIM from MCL-1 in these cells. Treatment with BIM SAHB_*A*_ sensitized DLBCL to ABT-737 by preventing BIM relocation onto MCL-1 following displacement from BCL-2. BIM SAHB_*A*_’s functional affinity for MCL-1 and induction of apoptosis at the level of the mitochondria was confirmed in MCL-1 deficient mouse embryonic fibroblasts (MEFs). This work highlights the importance of displacement and sequestration of BIM by anti-apoptotic BCL-2 proteins and further implicates a functional role of MCL-1 in apoptotic resistance in DLBCL. This study also illuminates the use of peptide-based BH3 mimetics to uncover biologically relevant cell death mechanisms and confirms that functional intracellular BH3-mimetic affinities for anti-apoptotic proteins may only partially reflect their acellular binding profiles.

## RESULTS

### Inverse correlation between DLBCL sensitivity to BIM SAHB_*A*_ and ABT-737/ABT-199

**Figure 1:**
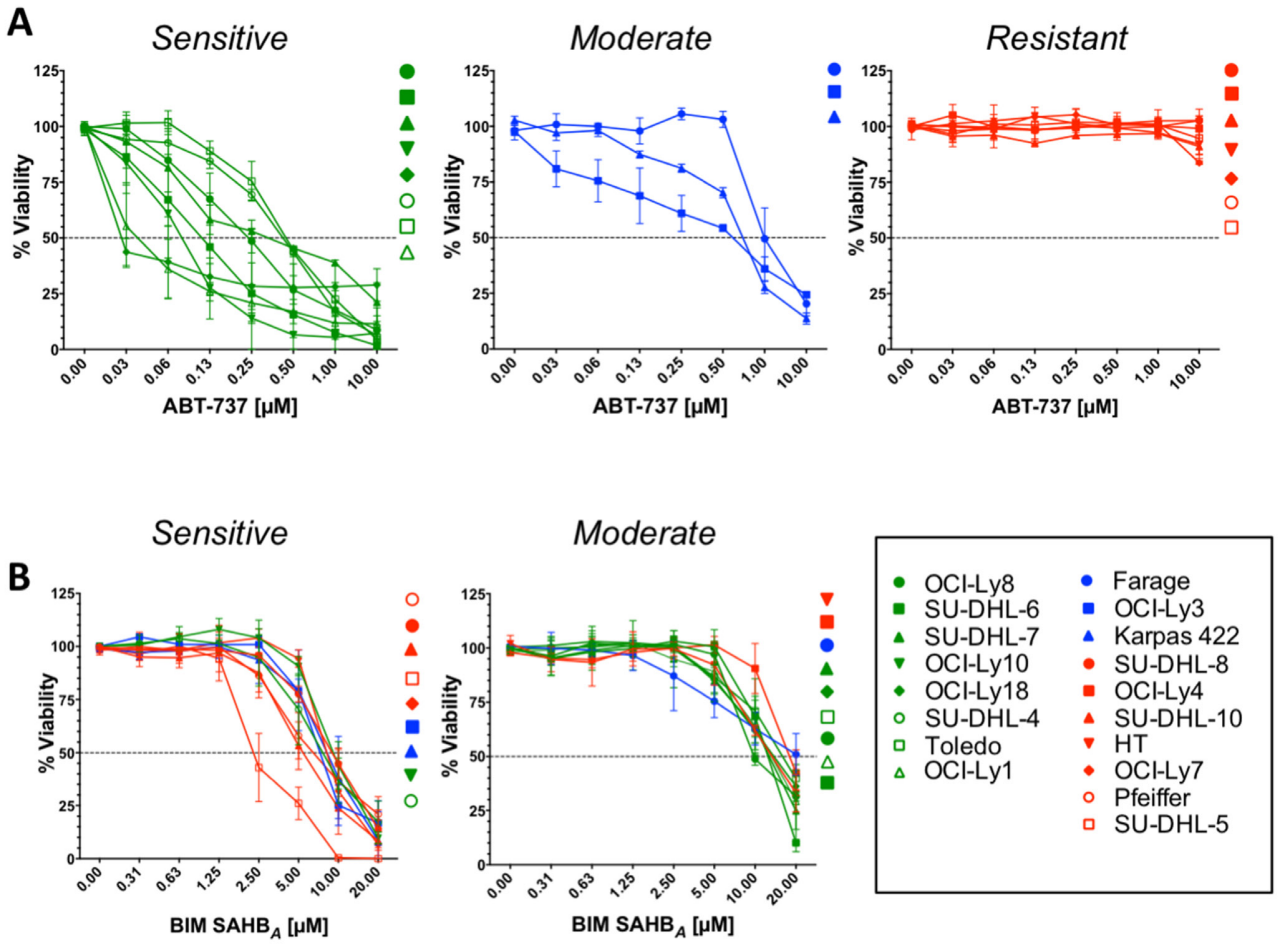
Sensitivity of DLBCLs to BIM SAHB_*A*_ inversely correlates with their sensitivity to ABT-737. Cell viability in a panel of human DLBCL cell lines was measured after 24-hr incubation with increasing concentrations of (**A**) ABT-737 or (**B**) BIM SAHB_*A*_. Percent (%) viability was calculated as a percentage of control (DMSO) treated cells. Dose-response curves indicating highest sensitivity to ABT-737 are in green, those indicating moderate sensitivity to ABT-737 are in blue, and those indicating low to no sensitivity to ABT-737 are in red. Error bars are mean ± SEM for at least three independent preparations of cell and BH3 mimetic treatments.

A panel of 18 human DLBCL cell lines was treated with increasing concentrations of BIM SAHB_*A*_, ABT-737, and ABT-199 to determine cell death as a result of distinctive anti-apoptotic targeting (Figure 1 and Supplementary Figure 1A). The cell lines were divided into 3 groups based on their ABT-737 sensitivity: ABT-737 sensitive, ABT-737 moderately sensitive, and ABT-737 resistant (Figure 1A). Our ABT-737 sensitivity profiles were similar to those previously reported using other measures of cell death, namely Annexin V positivity and ATP content [10, 11]. BCL-2 dependence in these cell lines was reflected in similar results following treatment with ABT-199 (Supplementary Figure 1A). Together, these results support increased BCL-X_L_ dependence in OCI-Ly10 and increased MCL-1 dependence in SU-DHL-10, HT, Pfeiffer, SU-DHL-5, SU-DHL-8, OCI-Ly7, and OCI-Ly4 cell lines as previously reported [10, 11, 17]. In contrast to treatment with ABT-737 and ABT-199, BIM SAHB_*A*_ induced dose-responsive cell death in all DLBCL cell lines with EC_50_’s ranging from 2 μM to 18 μM (Figure 1B and Supplementary Table 1). Like treatment with ABT-737 and ABT-199, DLBCL could be divided into two groups based on their sensitivities to BIM SAHB_*A*_: BIM SAHB_*A*_ ‘sensitive’ and BIM SAHB_*A*_ ‘moderately sensitive’ (Figure 1B). No death was measured in any cell line treated with a hydrocarbon-stapled BH3 point mutant control (BIM SAHB_*A*_ (R153D)) or vehicle alone indicating BIM-BH3 sequence-specific cell death induction (Supplementary Figure 1B and 1C) [16, 17, 19]. Based on our treatment analyses, there appeared to be an inverse correlation between DLBCL responses to ABT-737/ABT-199 and BIM SAHB_*A*_ (Supplementary Table 1).

### BIM SAHB_*A*_ induces caspase activation in DLBCL regardless of BCL-2 family protein expression

**Figure 2:**
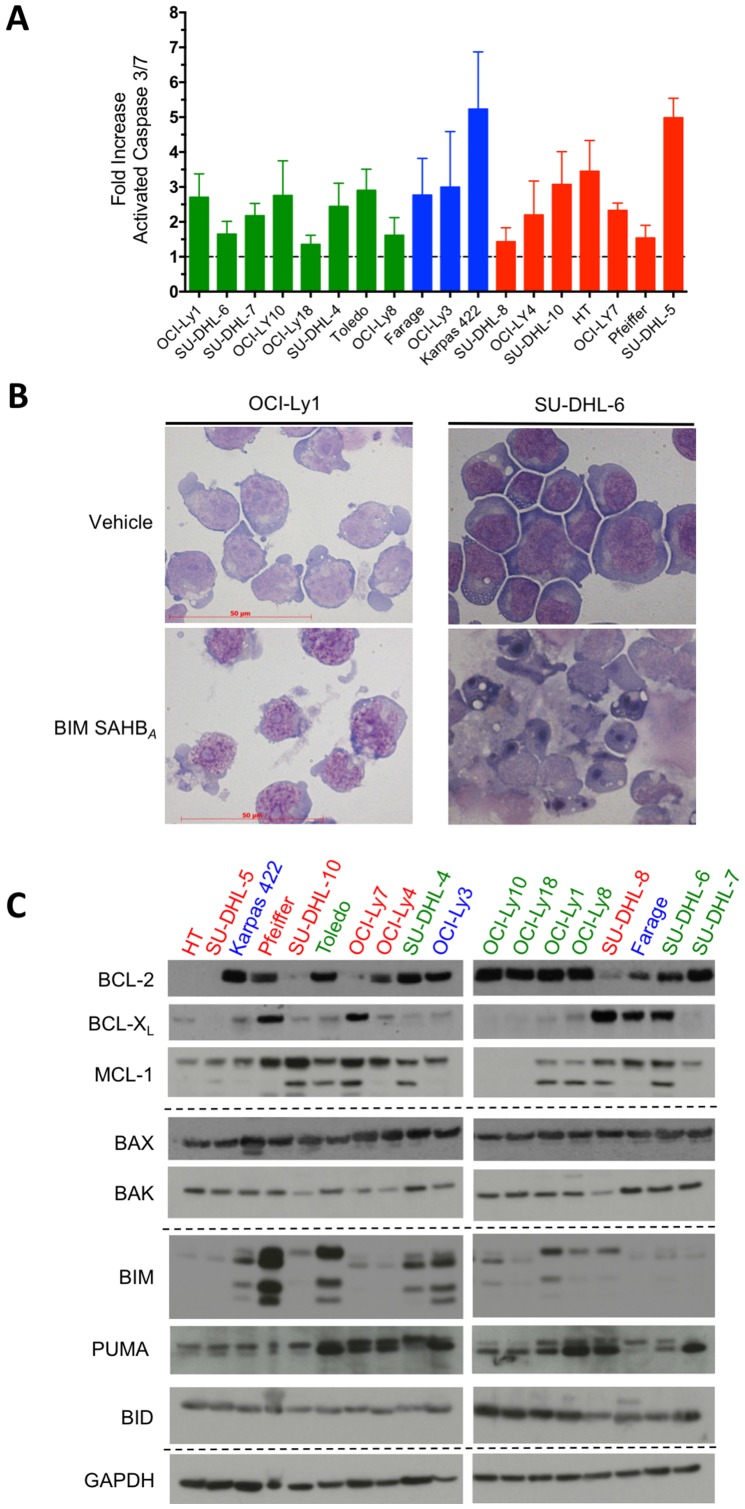
BIM SAHB_*A*_ treatment results in caspase 3/7 activation and cellular hallmarks of apoptosis irrespective of BCL-2 family protein expression in DLBCL. (**A**) DLBCL cell lines were treated BIM SAHB_*A*_ at their respective EC_50_ for 6-hr and activation of the intrinsic apoptotic pathway was assessed by monitoring the cleavage of pro-luminescent caspase 3/7 substrate. Caspase activity was calculated as fold change as compared to control (DMSO) treated cells. (**B**) BIM SAHB_*A*_-treatment results in hallmarks of apoptosis such as cellular membrane blebbing, cell shrinkage, chromatin marginalization, and nuclear fragmentation. (**C**) Western blot analysis of BCL-2 proteins in DLBCL cell lines. Colors of each cell line reflect their ABT-737 sensitivity patterns as depicted in Figure 1 and Supplementary Figure 1. Error bars are mean ± SEM for at least three independent preparations of cell and BIM SAHB_*A*_ treatments.

To confirm that BIM SAHB_*A*_ treatment lead to the activation of the intrinsic apoptotic pathway and MOMP, activated caspase 3/7 was measured six hours following treatment of DLBCL with BIM SAHB_*A*_ at their individual EC_50_ (Figure 2A). Cell death correlated with caspase 3/7 activation in all cell lines. The relative differences in caspase 3/7 activation between DLBCL may reflect differences in the kinetics of cell death in individual cell lines. Regardless, DLBCL partially or fully resistant to ABT-737 (shown in blue and red) had similar fold caspase activation compared to ABT-737 sensitive DLBCL (shown in green) (Figure 2A). Representative cell death morphology following treatment with BIM SAHB_*A*_ displayed classical hallmarks of apoptosis such as cellular membrane blebbing, cell shrinkage, chromatin marginalization, and nuclear fragmentation consistent with previous findings (Figure 2B) [17]. No correlation existed between BIM SAHB_*A*_-induced killing and protein expression levels of BCL-2, BCL-X_L_, or MCL-1 (Figure 2C and Supplementary Figure 2A–2C). This is similar to other reports where no correlations were measured between protein expression of BCL-2 and BCL-X_L_ and DLBCL responses to ABT-737 or ABT-199 [10, 11]. There was also no correlation in apoptosis and levels of other BCL-2 proteins known to be associated with cell death resistance in B-cell lymphomas including BIM, BID, PUMA, BAX, or BAK (Supplementary Figure 2D–2H) [10, 12, 20–23]. Given the importance of release of BIM for apoptosis induction in DLBCL, these results suggested that BIM SAHB_*A*_ was able to block endogenous BIM binding to MCL-1, BCL-2 and/or BCL-X_L_ expressing cells regardless of their sensitivity to ABT-737/ABT-199.

### BIM SAHB_*A*_ dissociates BIM from MCL-1 to a greater extent than BCL-2

**Figure 3:**
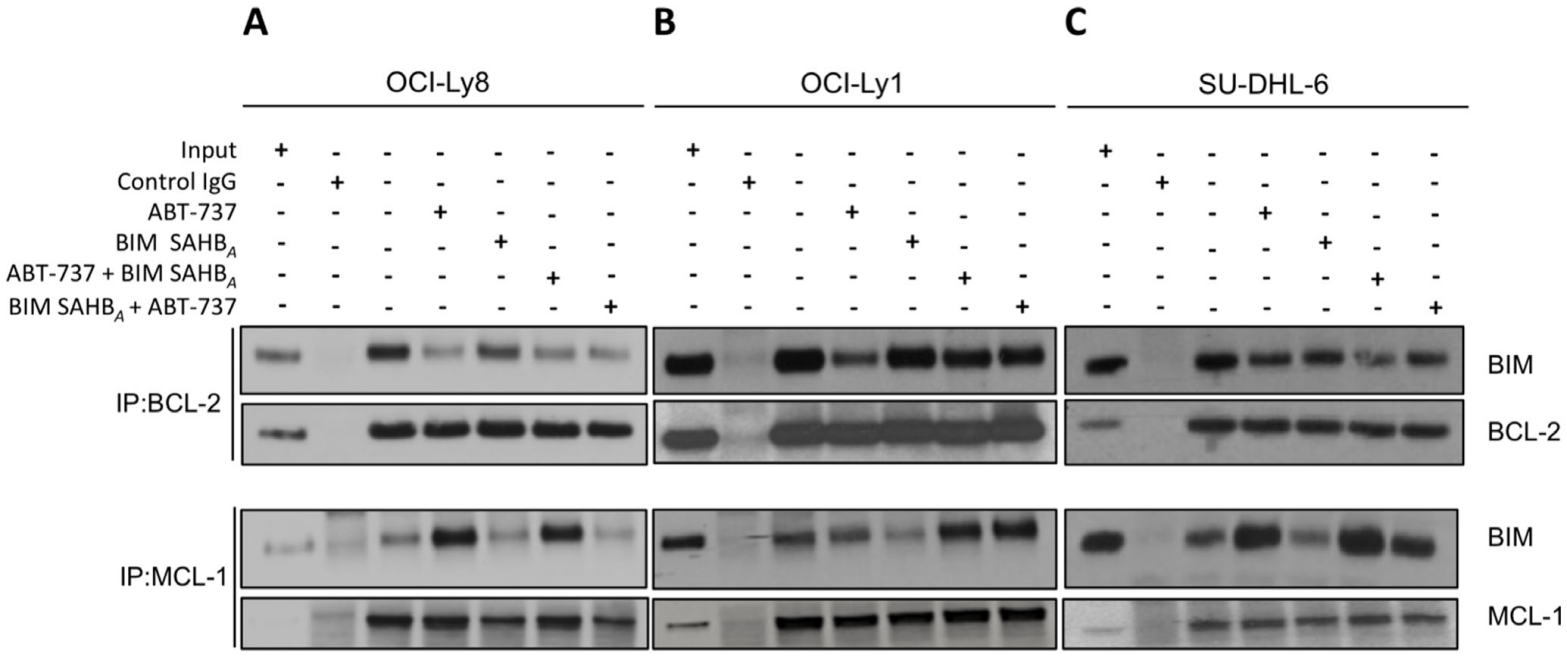
BIM SAHB_*A*_ preferentially targets MCL-1 over BCL-2 in ABT-737-sensitive DLBCL. (**A**) OCI-Ly8, (**B**) OCI-Ly1, and (**C**) SU-DHL-6 were left untreated (third lane) or treated with ABT-737 (EC_50_; fourth lane), BIM SAHB_*A*_ (EC_50_; fifth lane), ABT-737 for three hours followed by BIM SAHB_*A*_ for three hours (sixth lane), or BIM SAHB_*A*_ for three hours followed by ABT-737 for three hours (seventh lane). Lysates were immunoprecipitated with antibodies specific for BCL-2, MCL-1, or anti-Rabbit IgG (control), and immune complexes were resolved and immunoblotted for BIM, BCL-2 and MCL-1. Input lysate was loaded in the first lane and immunoprecipitates with a control IgG in the second lane. Treatments: OCI-Ly8: ABT-737 241 nM, BIM SAHB_*A*_ 13.2 μM; OCI-Ly1: ABT-737 30.7 nM, BIM SAHB_*A*_ 12 μM; SU-DHL-6: ABT-737 113 nM, BIM SAHB_*A*_ 12 μM.

We next sought to investigate the ability of BIM SAHB_*A*_ to release endogenous BIM from BCL-2 or MCL-1 in DLBCL that were sensitive (OCI-Ly1 > SU-DHL-6 > OCI-Ly8) or resistant (SU-DHL-5) to ABT-737 (Figure 1A, Supplementary Table 1) [10, 11]. The three ABT-737 sensitive cell lines were chosen as they represent an array of DLBCL phenotypes based on predominant anti-apoptotic BCL-2 family member expression (*e.g.* BCL-2, BCL-X_L_, and MCL-1). Importantly, these DLBCL depend, even if partially, on BCL-2, rather than BCL-X_L_ for cell death control [10, 13]. Treatment of individual cell lines with the EC_50_ of either ABT-737 or BIM SAHB_*A*_ alone was followed by immunoprecipitation of BCL-2 and MCL-1 and immunoblotting for endogenous BIM. As expected, treatment with ABT-737 resulted in dissociation of BIM from BCL-2 in BCL-2- expressing DLBCL (Figure 3). Once dissociated, BIM became sequestered by MCL-1, as measured particularly in OCI-Ly8 and SU-DHL-6 cells where the MCL-1:BIM complex following treatment with ABT-737 was greater than control treated cells (Figure 3A and 3C). In contrast, treatment with BIM SAHB_*A*_ led to measurable, but minimal, displacement of endogenous BIM from BCL-2 in comparison to ABT-737. BIM SAHB_*A*_ treatment resulted in much greater dissociation of BIM from MCL-1 with no detectable increased accumulation on BCL-2 in all three cell lines (Figure 3). Thus, although BIM SAHB_*A*_ was better able to dissociate endogenous BIM from MCL-1, it was still able to prevent redistribution of BIM to BCL-2 [17]. Given these results, we were interested if step-wise treatment with ABT-737 and BIM SAHB_*A*_ resulted in improved dissociation of BIM from either BCL-2 or MCL-1 and blockage of subsequent binding of released BIM on the alternate anti-apoptotic protein. To test this, cells were treated with either ABT-737 or BIM SAHB_*A*_ for three hours followed by addition of the other compound (*i.e.* ABT-737 + BIM SAHB_*A*_ or BIM SAHB_*A*_ + ABT-737). BIM SAHB_*A*_ was unable to release relocated BIM from MCL-1 that was dissociated from BCL-2 upon treatment first with ABT-737 (Figure 3). The increased BIM:MCL-1 association following treatment with ABT-737 overwhelmed the ability of BIM SAHB_*A*_ to effectively target MCL-1 at the same doses used during monotherapy. However, treatment with BIM SAHB_*A*_ before ABT-737 led to relocation of BIM from both BCL-2 and MCL-1. This phenomenon was greatest in OCI-Ly8 and SU-DHL-6 (Figure 3A and 3C) [10, 13]. In fact, although monotherapy of SU-DHL-5 and OCI-Ly1 with BIM SAHB_*A*_ led to release of endogenous BIM from MCL-1, this effect was largely lost in both combination treatments (Figure 3B and Supplementary Figure 3A) [24, 25]. There were no gross differences in expression of BCL-2, MCL-1, or BIM during the six hour treatment timeframe in any cell line following individual or combination treatments (Supplementary Figure 4). Dissociation of BAK from MCL-1 and activation of BAX in these cell lines may also have played a role in cell death following BIM SAHB_*A*_ treatment as has been reported previously [17, 26]. Dissociation of BAX from BCL-2 also occurs in these cell lines following treatment with both ABT-737 and BIM SAHB_*A*_ (Supplementary Figure 5).

### BIM SAHB_*A*_ sensitizes DLBCL to ABT-737

**Figure 4:**
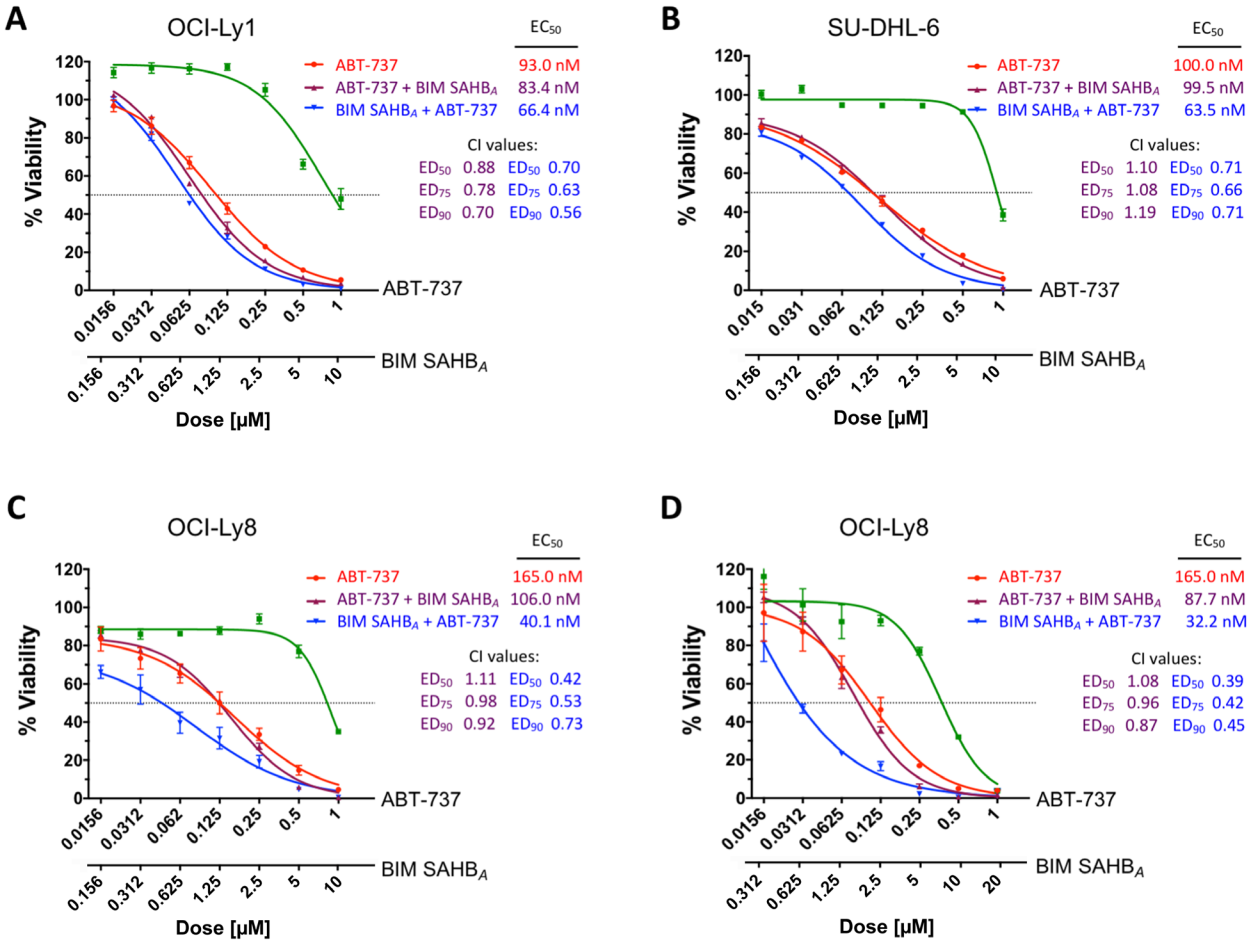
Treatment with BIM SAHB_*A*_ increases DLBCL cell death when given before but not following treatment with ABT-737. Cell viability of (**A**) OCI-Ly1, (**B**) SU-DHL-6, and (**C, D**) OCI-Ly8 was measured after 24-hr treatment with increasing concentrations of ABT-737 (red), BIM SAHB_*A*_ (green), ABT-737 for the first three hours followed by BIM SAHB_*A*_ (purple), or BIM SAHB_*A*_ for the first three hours followed by ABT-737 (blue). BIM SAHB_*A*_ given prior to ABT-737 universally led to a greater synergistic decrease in cell viability, as reflected by CalcuSyn analysis (combination index [CI] <1). Error bars are mean ± SEM for at least three independent preparations of cells and BH3 mimetic treatments. EC_50_, 50% effective concentration; ED_50_, 50% effective dose; ED_75_, 75% effective dose; ED_90_, 90% effective dose.

To test if sequential treatment would lead to differential effects on cell viability, DLBCL were treated as above and cell death was measured after 24 hours. Consistent with the BIM relocation results (Figure 3 and Supplementary Figure 3A) treatment with BIM SAHB_*A*_ prior to ABT-737 led to increased cell death when compared to the opposite treatment or monotherapy with either compound (Figure 4 and Supplementary Figure 3B). Equivalent results were measured when cells were treated with ABT-737 alone or with ABT-737 followed by BIM SAHB_*A*_ as was predicted by our BIM relocation data. The largest effect was measured in OCI-Ly8 correlating with BIM SAHB_*A*_’s ability to fully block BIM sequestration on MCL-1 following treatment with ABT-737 (Figure 4C). Regardless of overall effect, treatment with BIM SAHB_*A*_ prior to treatment with ABT-737 at a ratio of 10:1 universally led to increased cell death when compared to the reverse treatment in all cell lines (ABT-737 EC_50_: OCI-Ly8: 165 nM to 40.1 nM (76% decrease); OCI-Ly1: 93 nM to 66.4 nM (29% decrease); SU-DHL-6: 100 nM to 63.5 nM (37% decrease); and SU-DHL-5: >10,000 nM to 3,360 nM (~66% decrease) (Figure 4A–4C and Supplementary Figure 3B). In each case, synergy was observed when BIM SAHB_*A*_ was given prior to ABT-737, as reflected by combination indices of less than 0.8 at 50%, 75%, and 90% effective doses. Apart from synergy in OCI-Ly1, ABT-737 treatment prior to BIM SAHB_*A*_, predominantly resulted in an additive cell killing effect with combination indices between 0.8 and 1.1 [27]. These effects were magnified in OCI-Ly8 at a BIM SAHB_*A*_:ABT-737 ratio of 20:1 where the EC_50_ decreased from 165 nM to 32.2 nM (80% decrease) and synergy was increased at all effective doses indicating that the amount of BH3 mimetic may play a role in sequential dosing but does not significantly alter synergy values (Figure 4D). The increased sensitivity to ABT-737, particularly in OCI-Ly8, correlated with the ability of MCL-1 to absorb redistributed BIM once released from BCL-2 in these cells. Our results align with previous work showing that normal lymphoid cell and human non-Hodgkin lymphoma resistance to ABT-737 is conferred by mutant BIM specific for BCL-2, BCL-X_L_, and BCL-W [28]. However, mutant BIM specific for MCL-1 led to ABT-737 sensitivity indicating that ABT-737-mediated killing only occurred when MCL-1 was neutralized [28]. Our data would suggest that BIM SAHB_*A*_ is more selective for intracellular MCL-1 and effectively prevents BIM:MCL-1 association in a similar context.

To identify and correlate relevant BCL-2 pro-survival dependency in cells with either high (OCI-Ly8) or low (OCI-Ly1) MCL-1:BIM binding following ABT-737 treatment, we employed lentiviral expression of BIM_S_-derived BH3 variants [29]. These variants can determine biologically relevant BH3-only protein dependence within an intact cellular environment [30]. OCI-Ly8 and OCI-Ly1 were engineered to inducibly express: 1) BIM_S_WT able to target all pro-survival proteins, 2) BIM_S_2A able to target MCL-1 only, 3) BIM_S_BAD able to target BCL-2, BCL-X_L_ and BCL-W; or 4) BIM_S_4E unable to bind any anti-apoptotic, serving as a negative control (Supplementary Figure 6) [29]. BIM_S_WT expression greatly reduced the viability of both OCI-Ly8 and OCI-Ly1. OCI-Ly8 cells were more sensitive to BIM_S_BAD compared to BIM_S_2A indicating more BCL-2 dependence. This anti-apoptotic dependency pattern supports the increased cell death in OCI-Ly8 when treated first with BIM SAHB_*A*_ followed by ABT-737 where BIM, released from BCL-2 was unable to reoccupy itself on MCL-1 (Figure 4C). In contrast, targeting of MCL-1 by BIM_S_2A in OCI-Ly1 led to cell death equal to that of BIM_S_WT while the response to BIM_S_BAD was more moderate (Supplementary Figure 5B). Thus, OCI-Ly1 was overall more sensitive to BIM expression in this setting and more dependent on MCL-1 than BCL-2, supporting a different sequestration dynamic when endogenous BIM is dissociated from MCL-1 following BIM SAHB_*A*_ treatment. This is despite both DLBCL having similar baseline expression of BCL-2 (Supplementary Figure 2) [10, 11]. Together, these results support that the preferential intracellular target of BIM SAHB_*A*_ was MCL-1.

### MCL-1 is required for BIM SAHB_*A*_-dependent defects in the outer mitochondrial membrane

**Figure 5:**
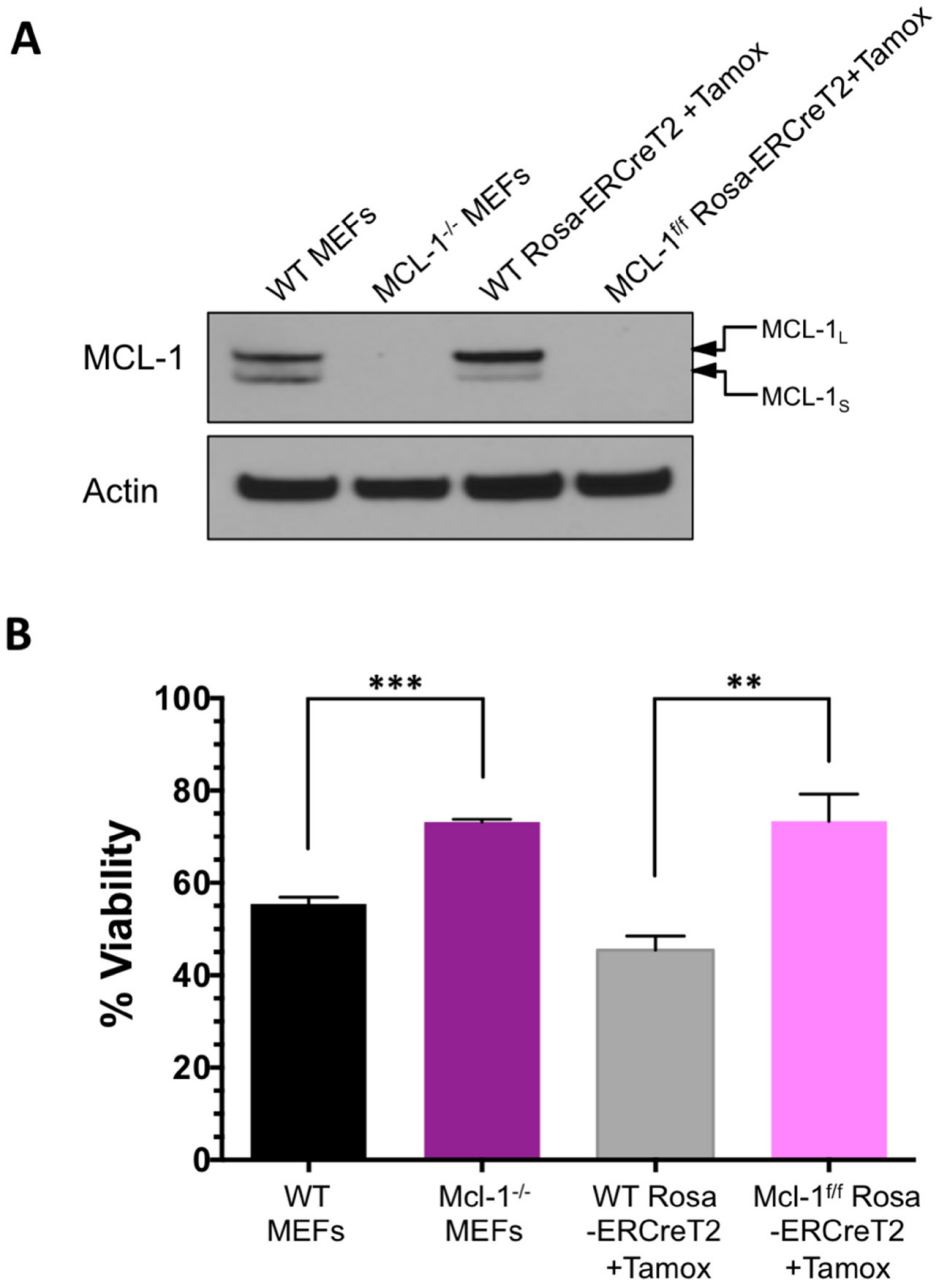
Absence of MCL-1 leads to resistance against BIM SAHB_*A*_-induced apoptosis. (**A**) Western blot analysis of MCL-1 expression in WT MEFs and MCL-1^-/-^ MEFs at steady-state and Rosa-ERCreT2 control MEFs and Mcl-1^*f/f*^ Rosa-ERCreT2 MEFs 72-hr following treatment with 100 nM of (4-hydroxy)-tamoxifen. Equivalent presence of MCL-1 was measured in the control MEFs and complete loss of MCL-1 was measured in MCL-1^-/-^ and Mcl-1^*f/f*^ Rosa-ERCreT2 MEFs. Short (S) and long (L) isoforms of MCL-1 are indicated by arrows. (**B**) Cell viability of these MEFs was measured following 24-hr treatment with 20 μM BIM SAHB_*A*_. Error bars are mean ± SEM for at least three independent preparations of cells and BH3 mimetic treatments.

Mouse embryonic fibroblasts (MEFs) are dependent upon MCL-1, and to a lesser extent, BCL-X_L_, for survival [31]. MCL-1 plays the more critical functional role in regulating apoptosis as reflected by the ability of ABT-737 to induce apoptosis only in MCL-1-deficient MEFs [9, 31]. We have previously shown that BIM SAHB_*A*_ can induce apoptosis in MCL-1-dependent and ABT-737-resistant malignant hematopoietic cells lines and can dissociate BAK from MCL-1 [17]. We have also shown that BIM SAHB_*A*_ can induce cell death in WT MEFs but not in BAX^-/-^BAK^-/-^MEFs [16, 17]. To investigate the general dependence on MCL-1 for BIM SAHB_*A*_-induced cell death we used MCL-1^-/-^ MEFs and MEFs with inducible deletion of MCL-1 (Mcl-1^*f/f*^ Rosa-ERCreT2 MEFs) to ensure there was no BCL-2 family compensation secondary to long-term MCL-1 loss [32]. WT and WT Rosa-ERCre T2 MEFs had equivalent expression of MCL-1 and MCL-1 protein was not detectable in either MCL-1^-/-^ or Mcl-1^*f/f*^ Rosa-ERCreT2 MEFs following treatment with tamoxifen (Figure 5A). Loss of MCL-1 in either MEF allowed for significant apoptosis in response to treatment with ABT-737 (Supplementary Figure 7). ABT-737 induced greater death in Mcl-1^*f/f*^ Rosa-ERCreT2 MEFs compared to MCL-1^-/-^ MEFs likely because MCL-1^-/-^ MEFs have developed BCL-2 compensatory mechanisms through long-term culturing as has been previously documented in genetic knockout model systems [33, 34]. In contrast to ABT-737, BIM SAHB_*A*_ induced death in both WT MEFs while either long-term or short-term absence of MCL-1 led to equivalent protection (Figure 5B). Only loss of MCL-1 was responsible for resistance to BIM treatment as indicated by equivalent cell death in MCL-1^-/-^ or Mcl-1^*f/f*^ Rosa-ERCreT2 MEFs.

**Figure 6:**
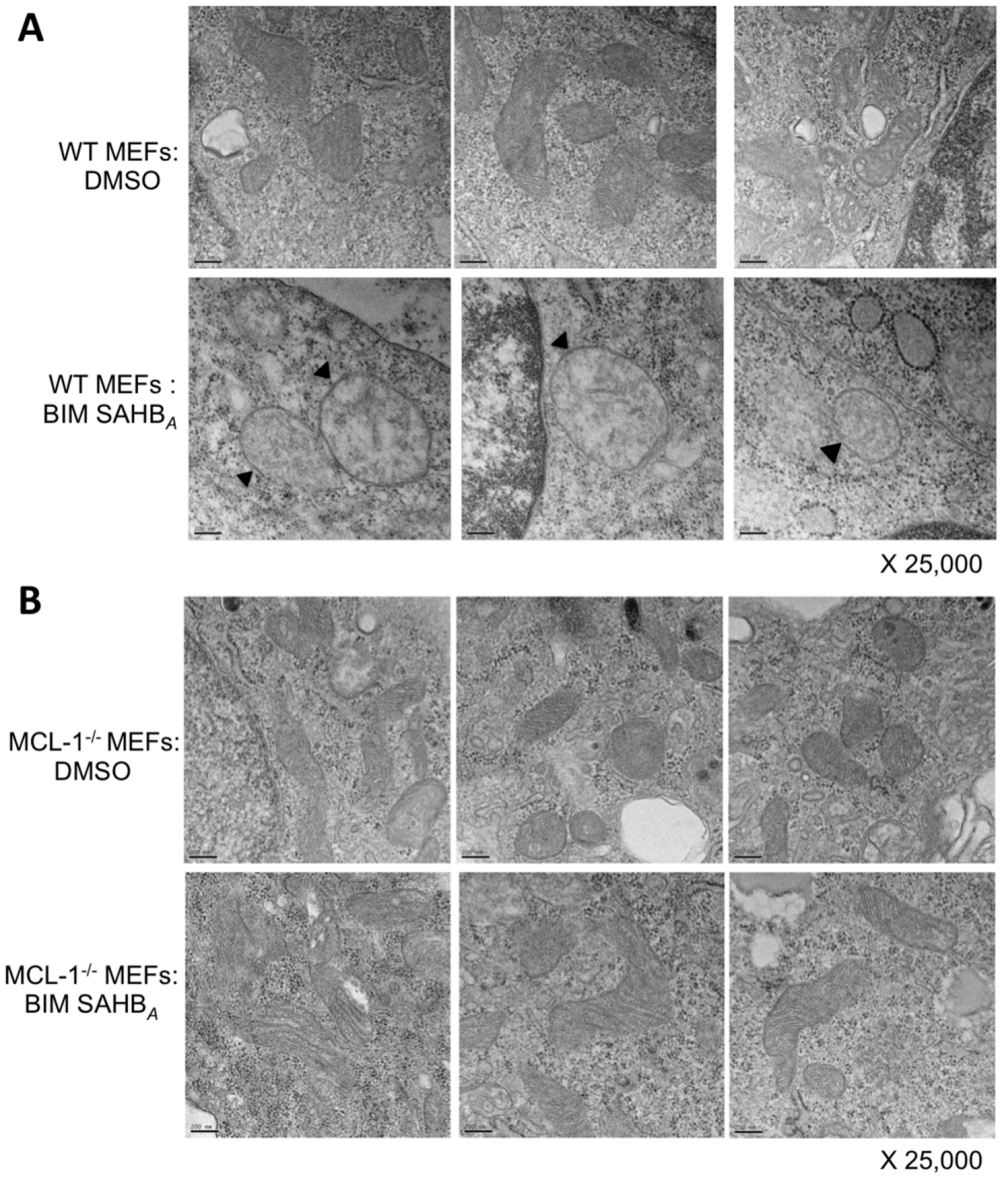
BIM SAHB_*A*_ induces characteristic hallmarks of apoptosis at the level of the mitochondria in the presence of MCL-1. (**A**) WT MEFs treated for 6-hr with DMSO (control; upper panels) or 20 μM BIM SAHB_*A*_ (lower panels) were monitored for mitochondrial apoptotic morphology using electron microscopy. (**B**) Identical treatment and imaging was performed on MCL-1^-/-^ MEFs. Black arrowheads point to mitochondrial swelling and loss of cristae architecture in BIM SAHB_*A*_-treated WT MEFs but not in MCL-1^-/-^ MEFS revealing a dependence on MCL-1 for BIM SAHB_*A*_-mediated apoptosis. x 25,000 magnification; scale bars, 200 nm.

We extended these results to investigate if treatment with BIM SAHB_*A*_ caused MCL-1-dependent changes in mitochondria morphology. Treatment of WT MEFs with BIM SAHB_*A*_ led to cristae disorganization and mitochondrial swelling, hallmarks of intrinsic apoptotic pathway activation (Figure 6A) [35, 36]. These phenotypic changes were not evident in treated MCL-1^-/-^ MEFs indicating that BIM SAHB_*A*_ preferentially targets MCL-1 in these cells (Figure 6B). These phenotypic data confirm BIM SAHB_*A*_’s ability to target intracellular MCL-1 and supports an MCL-1-dependent mechanism responsible for sensitivity to BIM SAHB_*A*_ [17].

## DISCUSSION

The presence of BCL-2 family anti-apoptotic members dictates cellular sensitivity to BH3 mimetics. However, expression alone is not enough to predict apoptotic functional dependency patterns, especially as it relates to malignant cell death. This is particularly evident in cells where expression of BCL-2, BCL-X_L_, or BCL-W does not always result in sensitivity to ABT-737 [37–41]. Additionally, *ex vivo* sensitivity against these agents does not always correlate with long-term *in vivo* results either in pre-clinical human xenograft studies or in patients [25, 39]. A primary reason for these resistance patterns is the up-front presence or emergence of MCL-1 during the course of treatment confirming the dynamism of the BCL-2 family regulatory network [42–44]. Importantly, despite the ability of ABT-737, or its oral analogue ABT-263, to bind BCL-2, BCL-X_L_, and BCL-W, it appears to overwhelmingly bind BCL-2 even when the other anti-apoptotics are present [28, 45]. A recent example of this preferential intracellular attraction is that treatment with ABT-199 or a BCL-X_L_-specific mimetic induced more cell death in BCL-2 and BCL-X_L_ expressing human small cell lung cancer (SCLC) and AML cell lines than treatment with ABT-263, indicating an intracellular preference for either BCL-2 or BCL-X_L_, but not both, by ABT-263 [46]. This phenomenon extends to *in vivo* testing in SCLC xenograft models where combination treatment with ABT-199 and the BCL-X_L_-specific mimetic resulted in better survival than treatment with ABT-263 alone [46]. Adding to the difficulty in navigating the BCL-2 targeting landscape, cancers derived from the same cell type can show different BCL-2 anti-apoptotic dependencies [10, 40, 41, 46, 47]. These differences often relate to levels of BIM expression and patterns of BIM:anti-apoptotic sequestration [47]. DLBCL is an example of such a disease where cell death resistance depends upon combinations of BIM bound to a number of antiapoptotics (BCL-2, BCL-X_L_, and MCL-1), and is therefore a useful model for testing the importance of both anti-apoptotic expression and BIM:anti-apoptotic sequestration differences relating to BH3-mimetic sensitivity [10–12, 25].

Like other BH3 mimetics, quantitative expression levels of BCL-2 proteins alone were not effective indicators of sensitivity to BIM SAHB_*A.*_ However, given the natural affinity of the BIM BH3 death domain to engage all anti- and pro-apoptotic BCL-2 proteins, it was not surprising that the cell permeable hydrocarbon-stapled BIM BH3 therapeutic reactivated cell death in all DLBCL tested. The ability of BIM SAHB_*A*_ to displace endogenous BIM from BCL-2 and MCL-1 supports the promise of the BIM BH3 domain as a therapeutic. Unexpectedly, like ABT-737/ABT-263 intracellular affinity for BCL-2 in many disease models, BIM SAHB_*A*_ has a propensity for targeting intracellular MCL-1 over BCL-2. This was likely responsible for its inverse sensitivity profile against the DLBCL cell lines compared to that of ABT-737 and ABT-199. In this regard, a therapeutic with BIM SAHB_*A*_’s therapeutic profile could be of particular benefit against activated B-cell like (ABC) DLBCLs that express *MCL1* at higher levels and have higher MCL-1:BIM binding than germinal center B-cell like (GCB) DLBCL [12]. However, despite the single agent efficacy of BIM SAHB_*A*_ against DLBCL as shown here, combination BH3 mimetic therapy will likely be necessary for reactivation of cell death at clinically useful levels [47–49]. Our data would also suggest that dosing and the sequence of such therapies are critical.

We found that BIM SAHB_*A*_ was unable to effectively dissociate a large amount of released endogenous BIM following treatment with ABT-737 when MCL-1 was present. However, prior to release from BCL-2, BIM SAHB_*A*_ was able to efficiently bind MCL-1 and prevent relocation of BIM in the more BCL-2-dependent and lower MCL-1 expressing cell lines (Figure 3A and 3C). It should be noted that DLBCL were treated at their EC_50_ in our study so as to capture cells before they underwent complete apoptosis. Additional studies will be needed to determine if increased doses of BIM SAHB_*A*_, as used in Figure 4D, or various treatment times would adequately remove BIM from DLBCL with larger amounts of MCL-1 following treatment with ABT-737 or ultimately stabilize MCL-1 protein levels as do newly introduced high-affinity small molecule MCL-1 inhibitors [6, 50]. In addition to dosing amounts, the timing of death induced by such treatments, either alone or in combination, likely differ between cell lines. Such differences could also be reflected in the relative amount of caspase 3/7 activation (Figure 2A) or synergy calculations in these and other cell lines where such combination treatments have been tested [11, 13, 14, 51]. Additionally, this work does not rule out the possibility of simultaneous triggering of caspase-dependent (*e.g.* apoptosome/caspase 3 and endonuclease activity) and caspase-independent (*e.g.* AIF) pathways following BIM SAHB_*A*_ treatment and caspase 3/7 activation. Despite this, the current study reinforces work indicating that the ability of BH3-mimetics to preferentially target intracellular BCL-2 anti-apoptotic members often does not completely reflect their *in vitro* binding profile(s) [4, 10, 28, 45]. We and others have shown that BIM SAHB_*A*_ binds MCL-1, BCL-X_L_, BCL-2, and A1 with low nanamolar affinity and is able to dissociate BAK and BAX BH3 ligands from MCL-1 and BCL-X_L_ respectively [17, 52]. Although able to bind all anti-apoptotics tested, the current study supports that the preferential intracellular target of BIM SAHB_*A*_ is MCL-1 over BCL-2, at least in DLBCL. However, we cannot discount that direct activation of BAX or dissociation of other BH3-only proteins, such as BID, plays a role in the overall cell death effectiveness of BIM SAHB_*A*_ in these same cells [16, 17, 26, 52].

Well-timed combination BH3 mimetic therapy may be a promising strategy against diseases like DLBCL where oncoproteins (*e.g.* MYC, BCL6, and BCL-2) play roles in blocking the intrinsic apoptotic network through direct upregulation of BCL-2 family members and other cell death-related proteins [53, 54]. Such complex perturbation in apoptotic regulation necessitates a combinatorial therapeutic approach involving multiple BH3 mimetics, which may include small molecule and peptide-based therapeutics, alone or with other conventional chemotherapies. A complete understanding of intracellular affinities and the therapeutic ramifications of targeted anti-apoptotic therapeutics on relocation of BIM will be critical as BH3-mimetic therapies are advanced.

## MATERIALS AND METHODS

### Cell culture and therapeutic reagents

Human DLBCL cell lines (kind gift from Margaret A. Shipp, M.D., Dana-Farber Cancer Institute, Boston, MA 02115, USA and purchased from ATCC and DSMZ) were cultured in RPMI 1640 medium supplemented with 0.5 mg/ml penicillin/streptomycin, 2 mM L Glutamine, 1 mM HEPS, Non-Essential Amino Acids (all from Life technologies) and 10% heat inactivated fetal bovine serum (*Denville Scientific*). HEK293T were obtained from American Type Culture Collection. HEK293T cells and MEFs Cells were grown in Dulbecco’s modified Eagle’s medium (DMEM, Life technologies) supplemented with same reagents as previously mentioned. For BIM SAHB_*A*_ treatment, cells were cultured in advanced RPMI supplemented with 0.5 mg/ml penicillin/streptomycin, 2 mM L Glutamine, 1 mM HEPS, 1% NEAA. All cells were maintained at 37°C in 5% CO_2_. Wild-type ERCreT2 and Mcl-1f/f Rosa-ERCreT2 MEFs were a kind gift from Joseph T. Opferman Ph.D., St. Jude Children’s Research Hospital. BIM SAHB_*A*_ and BIM SAHB_*A*_ (R153D) were synthesized, purified, as previously reported [16, 17, 19]. SAHB constructs used in caspase assays and confocal cell death assays were a kind gift from Loren D. Walensky, M.D., Ph.D. and Gregory Bird, Ph.D., Dana-Farber Cancer Institute, Boston, MA. All other SAHBs used for cell treatments were synthesized at the University of Chicago. Doxycycline (Sigma), ABT-199 (Selleck Chemicals), ABT-737 (Selleck Chemicals), (4-hydroxy)-tamoxifen (Sigma).

### Viability assays

DLBCL (2 × 10^4^ cells) were treated with serial dilutions of BIM SAHB_*A*_, ABT-199, ABT-737 or DMSO for 2 h in serum free advanced RPMI media followed by addition of FBS to the media (to 10% [v/v]) as previously described [17]. Viability was assessed using Cell proliferation kit II XTT (Roche) according to the manufacturer’s instructions. Absorbance EC_50_s were calculated using GraphPad Prism 6 software. For combination treatments, DLBCL (2 × 10^4^ cells) were treated with serial dilutions of BIM SAHB_*A*_ (0–20 μM) or ABT-737 (0–1 or to 20 μM), 3 h in serum free advanced RPMI media followed by 3 h of treatment with either ABT-737, BIM SAHB_*A*_ or DMSO (0.2%). FBS was then added to the media (to 10% [v/v]). Viability was assessed 24 h later using CellTiter-Glo according to the manufacturer’s instructions. (XTT) or luminescence (CellTiter-Glo) was detected by Synergy 2 microplate reader (BioTek). Synergy analyses were performed as previously described [17].

### Caspase-3/7 activation assay

DLBCL (10,000 cells/well) were seeded in 96-well plates in advanced RPMI Growth Media and were treated with BIM SAHB_*A*_ or DMSO for 6 hours. Caspase-Glo 3/7 chemiluminescence reagent (Promega) was used according to manufacturer’s directions. Luminescence was measured by a Synergy 2 microplate reader (BioTek) and was standardized to DMSO treated samples as previously described [17].

### Western blot analysis

Following treatment of individual DLBCL with compounds, cells were washed with PBS and lysed in cell lysis buffer (25 mM HEPES pH 7.5, 150 mM NaCl, 1% Triton X-100, 0.1% SDS, 5 mM EDTA and 1 mM NaF supplemented with 1x protease inhibitor cocktail tablets [Roche]) as previously described [17]. Protein concentrations were determined by Pierce BCA Protein Assay Kit (Thermo Scientific). 30 μg of protein were electrophoretically separated on NuPAGE 12% Bis-Tris polyacrylamide gels (Invitrogen). Separated proteins were transferred to nitrocellulose membranes (BioRad). Blots were incubated with the following antibodies: MCL-1 (Santa Cruz, S-19), BCL-2 (Epitomics, 1017-1), BCL-X_L_ (Santa Cruz, S18), BIM (Millipore AB17003), BAX (Santa Cruz, N20), BAK (Millipore 06-536), BID (Santa Cruz), GAPDH (Santa Cruz FL-335), PUMA (Santa Cruz). The immune complex was detected using anti-rabbit HRP-conjugated antibody (Envision Detection Kit, DakoCytomation) and the chemiluminescent detection kit according to the manufacturer’s specifications (Amersham). Proteins were visualized with *CL-XPosure Film* (Thermo Scientific) using AX200 X-Ray film processor (Aphatek).

### Immunoprecipitation

DLBCL (9 × 10^6^ cells) were lysed in 2 ml of Triton X-100 buffer (50 mM Tris-HCL [pH 7.4], 150 mM NaCl, 5 mM MgCl_2_, 1 mM EGTA, 10% Glycerol, 1% Triton X-100 and 1x protease inhibitor cocktail tablets [Roche]) on ice for 30 min and then centrifuged at 18,000 × g for 20 min at 4°C. 400 μg proteins was pre-cleared using 30 μl of Pierce Protein A Magnetic Beads (Thermo Scientific) for 2 hours at 4°C then incubated overnight at 4°C with MCL-1 antibody (Protein Tech, 16225), BCL-2 antibody (Epitomics, 1017–1) or Rabbit IgG. 20 μl of Magnetic beads were added and incubated for 2 hours. Immunoprecipitates were then washed 5 times with 500 μl of Triton X-100 buffer and boiled in *NuPAGE LDS Sample Buffer* (Thermo Scientific) supplemented with 1 mM DTT and proteins were separated on NuPAGE 10% Bis-Tris polyacrylamide gels.

### Wright-giemsa stain

DLBCL cells were treated with vehicle or BIM SAHB_*A*_ in Opti-MEM media for 4 hours at 37°C. Cells stained as previously described [17]. Briefly, cells were collected and washed twice with PBS containing 2% FBS and resuspended at 2.5 × 10^6^ cells/ml for mounting of the cellular suspension onto glass slides by cytospin. The slides were air dried and then stained with Accustain Modified Wright-Giemsa according to the manufacturer’s protocol (Sigma-Aldrich).

### BIM_s_ isoform lentivirus production and transduction

The TRIPZ vectors with inducible expression of the different variants of BIM_s_ were a kind gift from Andreas Strasser, Ph.D. (The Walter and Eliza Hall Institute, Parkville, Melbourne) [29]. To generate cells stably expressing the inducible BIM_s_ variants, DLBCL cells were infected with viral supernatants, produced by lipofectamine 2000 co-transfection of 293T cells with TRIPZ expression constructs and packaging plasmids according to the manufacture protocol and as previously described [29]. Cells were expanded then FACS-sorted for the expression of GFP using a *BD FACSAria* cell sorter. The expression of BIMs proteins was induced as previously described [29].

### Electron microscopy

Indicated MEFs were plated in six-well format (2 × 10^5^ cell/condition) were washed in serum free Advanced DMEM following overnight induction. The cells were then incubated with 20 μM BIM SAHB_*A*_ or vehicle (0.05% [v/v] DMSO) for 2-hr, and then FBS was added for an additional 4-hr. The cells were washed twice with advanced DMEM media and fixed in the dish using a solution of 2% (w/v) glutaraldehyde, 4% (w/v) paraformaldehyde, in 0.1M sodium cacodylate buffer overnight at RT. The cells were then washed in 0.1 M sodium cacodylate buffer (pH 7.4) 3X5 min, post-fixed for 60 min in 1% (w/v) 1% Osmium Tetroxide in 0.1 M sodium cacodylate buffer, washed in sodium cacodylate buffer 2X5 min and Maleate buffer (pH5.1) once, 5 min, and then incubated in 1% (w/v) aqueous uranyl acetate in Maleate buffer for 60 min, followed wash in Maleate buffer 3X5 min. The cells were removed from the dish in 2:1 propylene oxide and incubated overnight in a 1:1 mixture of propylene oxide. The samples were subsequently embedded in TAAB Epon and polymerized at 60°C for 48 h. Ultrathin sections (~90 nm) were cut on a Reichert-Jung Ultracut E microtome, placed onto copper grids, stained with Uranyl acetate and Lead citrate, and then examined under 300KV at FEI Tecnai F30 and the Gatan CCD digital micrograph. Electron microscopic sample preparation and imaging was performed in conjunction with the Advanced Electron Microscopy facility at the University of Chicago.

## SUPPLEMENTARY MATERIALS



## Abbreviations

ABC: activated B-cell
CCD: charge-coupled device
DLBCL: diffuse large B-cell lymphoma
DMEM: Dulbecco’s modified Eagle medium
DMSO: dimethyl sulfoxide
DTT: dithiothreitol
EC50: half-maximal concentration of drug
EGTA: ethylene glycol-bis(β-aminoethyl)-N,N,N’,N’-tetraacetic acid
FACS: fluorescence-activated cell sorter
GCB: germinal center B-cell
GFP: green-fluorescent protein
HEPES: 4-(2-hydroxyethyl)-1-piperazineethanesulfonic acid
Hr: hour
MEF: mouse embryonic fibroblast
Min: minute
mM: millimolar
MOMP: mitochondrial outer membrane permeabilization
NEAA: non-essential amino acids
nM: nanomolar
PAGE: polyacrylamide gel electrophoresis
PBS: phosphate buffered saline
RPMI: Roswell Park Memorial Institute medium
RT: room temperature
SDS: sodium dodecyl sulfate
SEM: standard error of the mean
μM: micromolar
WT: wild-type
