# Supplementary material for: Preferential targeting of MCL-1 by a hydrocarbon-stapled BIM BH3 peptide
Source: Oncotarget. 2019 Oct 22;10(58):6219–33. doi: 10.18632/oncotarget.27262 (PMC6817437; doi:10.18632/oncotarget.27262)
Supplement: Supplementary file 1 [file oncotarget-10-6219-s001.pdf]

Preferential targeting of MCL-1 by a hydrocarbon-stapled BIM BH3 peptide

SUPPLEMENTARY MATERIALS

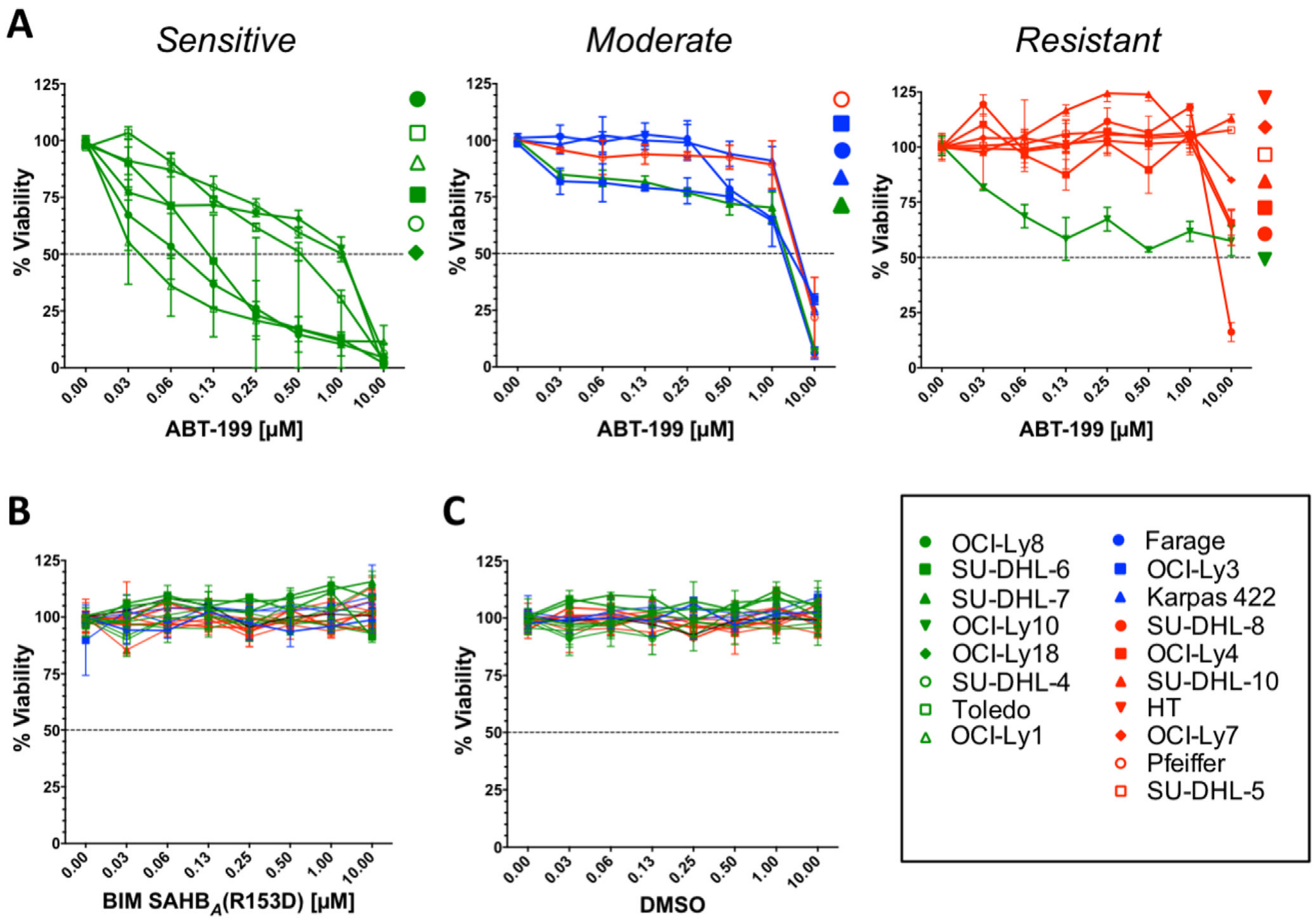

Supplementary Figure 1: Sensitivity of DLBCLs to BIM SAHB$_A$ inversely correlates with their sensitivity to venetoclax (ABT-199). Cell viability in a panel of human DLBCL cell lines was measured after 24-hr incubation with increasing concentrations of (**A**) ABT-737, (**B**) BIM SAHB$_A$ R153D, or (**C**) equivalent concentrations of DMSO. Percent (%) viability was calculated as a percentage of control (DMSO) treated cells. Dose-response curves indicating highest sensitivity to ABT-737 are in green, those indicating moderate sensitivity to ABT-737 are in blue, and those indicating low to no sensitivity to ABT-737 are in red. Error bars are mean ± SEM for at least three independent preparations of cell and BH3 mimetic treatments.

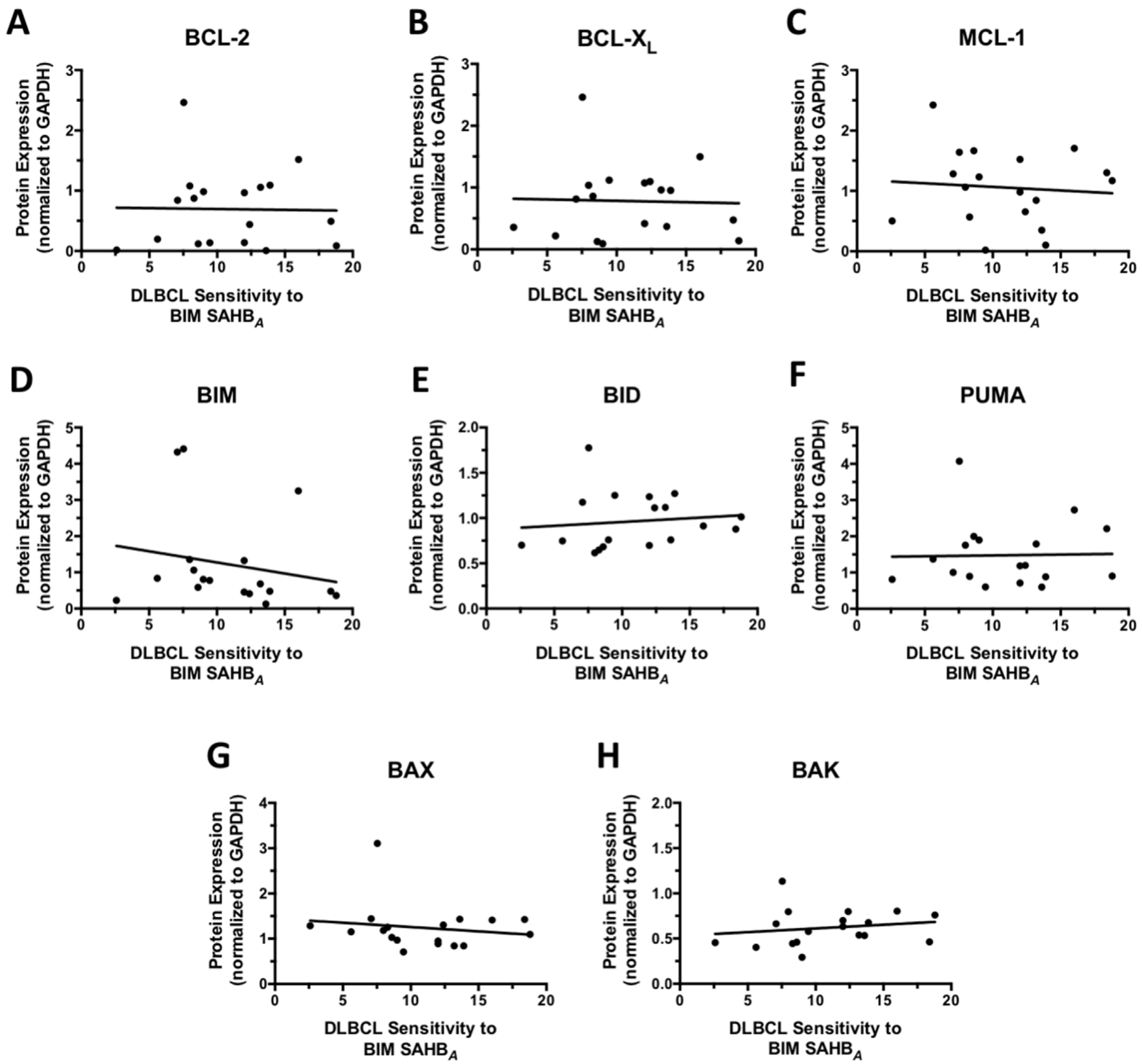

Supplementary Figure 2: Individual BCL-2 family protein expression in DLBCL does not correlate with BIM SAHB$_A$ sensitivity. BCL-2 family proteins in DLBCL analyzed by immunoblotting were statistically analyzed via a linear regression model. X-axis is EC_{50} of BIM SAHB$_A$ [μM] for each cell line; Y-axis represents densitometric units of specific BCL-2 proteins as indicated, obtained by densitometric analysis of immunoblots. All R^2 are non-significant with $P > 0.05$.

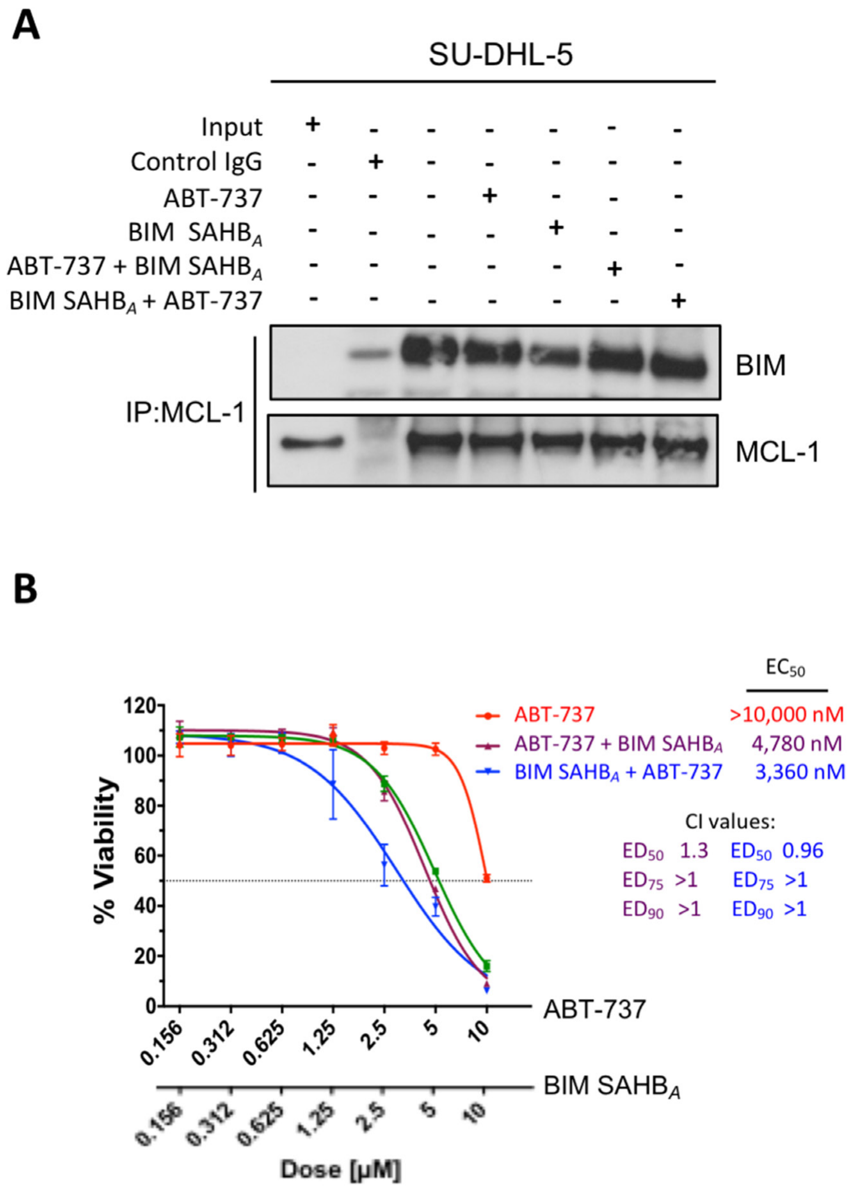

Supplementary Figure 3: BIM SAHB$_A$ targets MCL-1 in ABT-737-resistant DLBCL. (**A**) SU-DHL-5 were left untreated (third lane) or treated with ABT-737 (EC$_{50}$; fourth lane), BIM SAHB$_A$ (EC$_{50}$; fifth lane), ABT-737 for three hours followed by BIM SAHB$_A$ for three hours (sixth lane), or BIM SAHB$_A$ for three hours followed by ABT-737 for three hours (seventh lane). Lysates were immunoprecipitated with antibodies specific for BCL-2, MCL-1, or anti-Rabbit IgG (control), and immune complexes were resolved and immunoblotted for BIM, BCL-2 and MCL-1. Input lysate was loaded in the first lane, immunoprecipitates with a control IgG in the second lane, and no treatment controls in the third lane. Treatments: SU-DHL-5: ABT-737 10 μM, BIM SAHB$_A$ 2.6 μM (**B**) Cell viability of SU-DHL-5 was measured after 24-hr treatment with increasing concentrations of ABT-737 (red), BIM SAHB$_A$ (green), ABT-737 for the first three hours followed by BIM SAHB$_A$ (purple), or BIM SAHB$_A$ for the first three hours followed by ABT-737 (blue). Error bars are mean ± SEM for at least three independent preparations of cells and BH3 mimetic treatments.

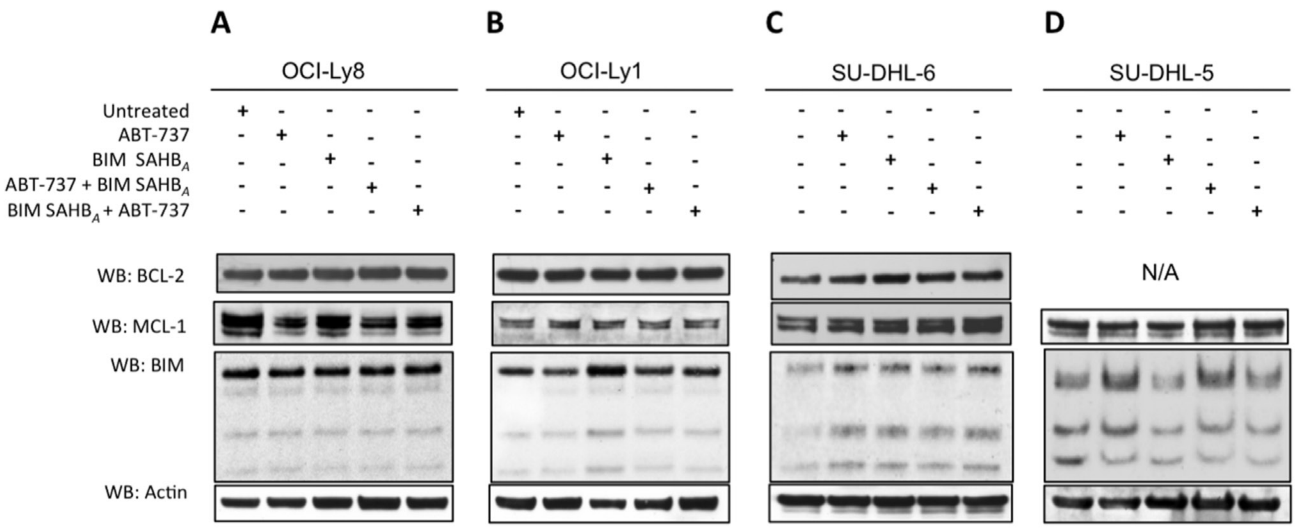

Supplementary Figure 4: Treatment of DLBCL with ABT-737, BIM SAHB$_A$, or combination of each do not change BCL-2, MCL-1, or BIM protein expression. BCL-2, MCL-1, and BIM expression as detected by Western blot analysis of cellular lysates from (**A**) OCI-Ly8, (**B**) OCI-Ly1, (**C**) SU-DHL-6, and (**D**) SU-DHL-5 following treatment with ABT-737, BIM SAHB$_A$, or combinations of the two do not grossly differ from each other or from untreated cells. Treatments: OCI-Ly8: ABT-737 241 nM, BIM SAHB$_A$ 13.2 µM; OCI-Ly1: ABT-737 30.7 nM, BIM SAHB$_A$ 12 µM; SU-DHL-6: ABT-737 113 nM, BIM SAHB$_A$ 12 µM; SU-DHL-5: ABT-737 10 µM, BIM SAHB$_A$ 2.6 µM.

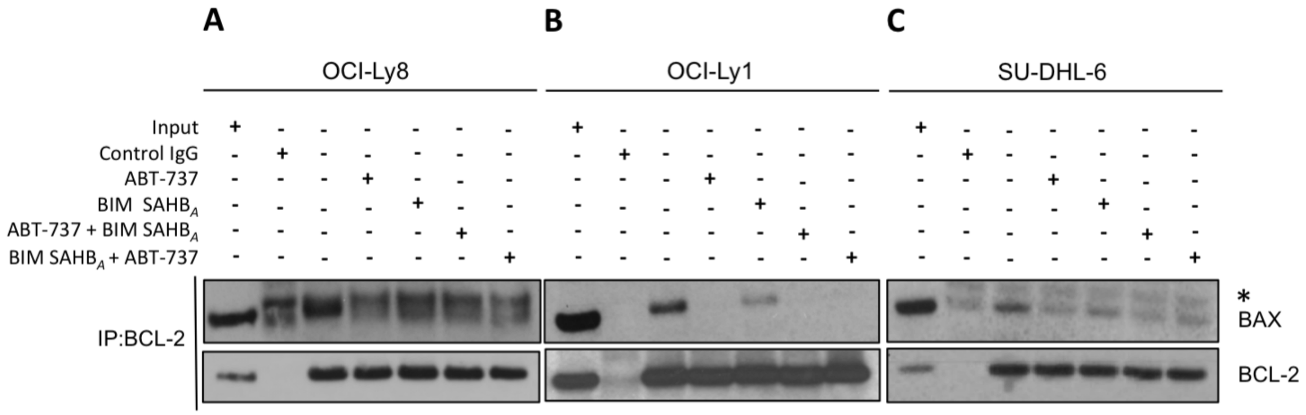

Supplementary Figure 5: BAX is dissociated from BCL-2 following treatment with ABT-737 alone or in combination with BIM SAHB$_A$. (**A**) OCI-Ly8, (**B**) OCI-Ly1, and (**C**) SU-DHL-6 were left untreated (third lane) or treated with ABT-737 (EC_{50}; fourth lane), BIM SAHB$_A$ (EC_{50}; fifth lane), ABT-737 for three hours followed by BIM SAHB$_A$ for three hours (sixth lane), or BIM SAHB$_A$ for three hours followed by ABT-737 for three hours (seventh lane). Lysates were immunoprecipitated with antibodies specific for BCL-2 or anti-Rabbit IgG (control), and immune complexes were resolved and immunoblotted for BAX and BCL-2. Input lysate was loaded in the first lane and immunoprecipitates with a control IgG in the second lane. Treatments: OCI-Ly8: ABT-737 30.7 nM, BIM SAHB$_A$ 13.2 µM; OCI-Ly1: ABT-737 241 nM, BIM SAHB$_A$ 12 µM; SU-DHL-6: ABT-737 113 nM, BIM SAHB$_A$ 12 µM. *non-specific band.

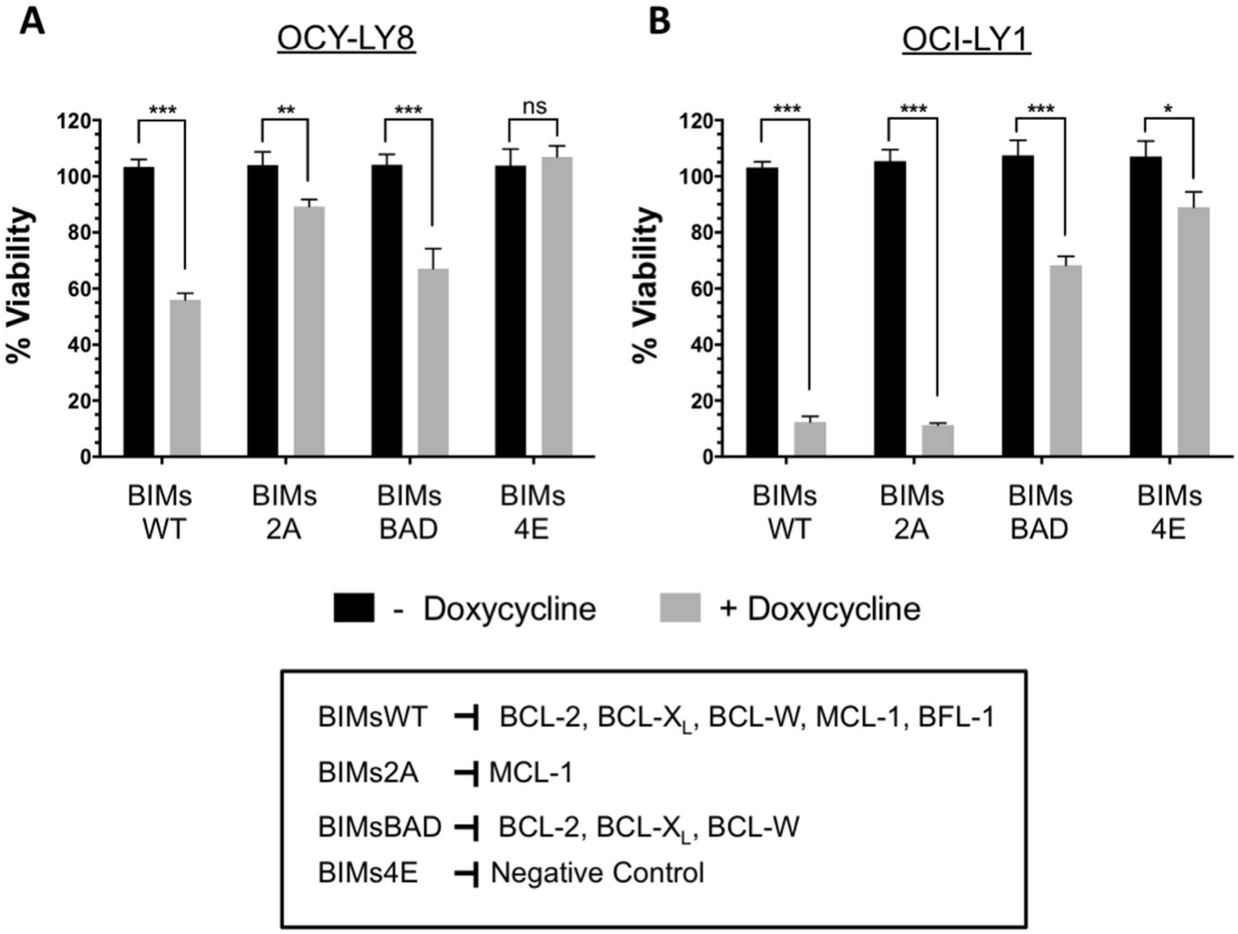

Supplementary Figure 6: BIM$_s$ BH3 variants that selectively neutralize specific anti-apoptotic BCL-2 proteins define BCL-2 or MCL-1 dependence in DLBCL. Cell viability of (**A**) OCI-Ly8 cells and (**B**) OCI-Ly1 cells transduced with various inducible lentiviral BIM$_S$ constructs was measured 24-hr following treatment with doxycycline. Using this method, both cells are dependent on BCL-2 and MCL-1 but have different 'main' dependences: OCI-Ly8 (BCL-2>MCL-1) and OCI-Ly1 (MCL-1>BCL-2). Error bars are mean ± SEM for at least three independent experiments. ($^{*}P < 0.05$; $^{**}P < 0.01$, $^{***}P < 0.001$).

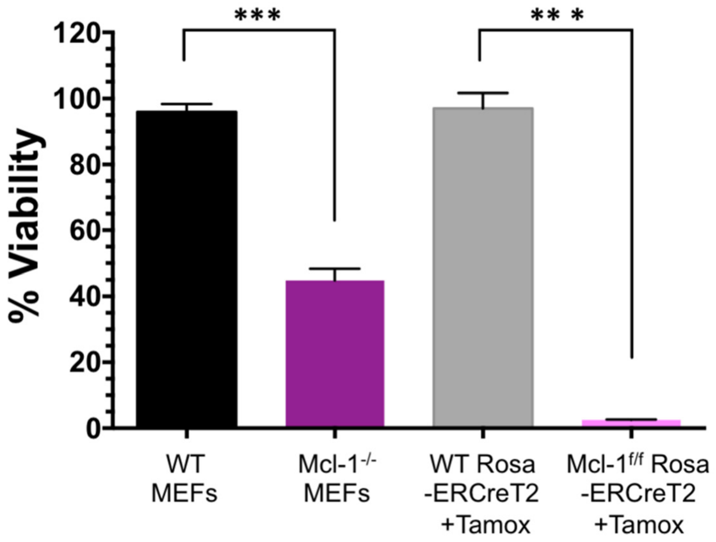

Supplementary Figure 7: Loss of MCL-1 promotes MEF susceptibility to ABT-737-mediated apoptosis. Cell viability of WT MEFs, native MCL-$1^{-/-}$ MEFs, Rosa-ERCreT2 MEFs, and Mcl-$1^{f/f}$ Rosa-ERCreT2 MEFs was measured 24-hr following treatment with ABT-737 (500 nM). Error bars are mean ± SEM for at least three independent experiments. ($^{*}P < 0.05$; $^{**}P < 0.01$, $^{***}P < 0.001$).

Supplementary Table 1: EC_{50} of ABT-199, ABT-737 and BIM $SAHB_A$ in DLBCL cell lines

ABT-737		ABT-199		BIM $SAHB_A$	
**Cell Line**	**EC_{50} [M]**	**Cell Line**	**EC_{50} [M]**	**Cell Line**	**EC_{50} [M]**
OCI-Ly18	9.14E-10	OCI-Ly1	4.32E-09	SU-DHL-5	2.59E-06
OCI-Ly1	3.07E-08	OCI-Ly8	7.11E-08	SU-DHL-10	5.61E-06
OCI-Ly10	7.90E-08	SU-DHL-6	1.22E-07	Pfeiffer	7.08E-06
SU-DHL-6	1.13E-07	Toledo	4.51E-07	OCI-Ly3	7.54E-06
OCI-Ly8	2.41E-07	OCI-Ly18	7.00E-07	SU-DHL-4	7.98E-06
SU-DHL-4	4.04E-07	SU-DHL-4	7.84E-07	Karpas 422	8.29E-06
SU-DHL-7	4.28E-07	Farage	1.45E-06	OCI-Ly7	8.61E-06
Toledo	4.63E-07	SU-DHL-7	1.48E-06	SU-DHL-8	8.99E-06
OCI-Ly3	5.55E-07	OCI-Ly3	2.73E-06	OCI-Ly10	9.46E-06
Karpas 422	6.75E-07	Pfeiffer	3.93E-06	OCI-Ly1	1.20E-05
Farage	1.00E-06	Karpas 422	4.70E-06	SU-DHL-6	1.20E-05
SU-DHL-5	>1.0E-05	SU-DHL-8	8.06E-06	SU-DHL-7	1.24E-05
OCI-Ly7	>1.0E-05	OCI-Ly10	>1.0E-05	OCI-Ly8	1.32E-05
HT	>1.0E-05	SU-DHL-5	>1.0E-05	HT	1.36E-05
SU-DHL-10	>1.0E-05	OCI-Ly7	>1.0E-05	OCI-Ly18	1.39E-05
OCI-Ly4	>1.0E-05	HT	>1.0E-05	Toledo	1.60E-05
SU-DHL-8	>1.0E-05	SU-DHL-10	>1.0E-05	OCI-Ly4	1.84E-05
Pfeiffer	>1.0E-05	OCI-Ly4	>1.0E-05	Farage	1.88E-05

Sensitivity of DLBCLs to BIM $SAHB_A$ inversely correlates with their sensitivity to ABT-737 and ABT-199. Summary of the EC_{50}s of the different compounds in DLBCL cell lines ranked from lowest to highest EC_{50}.
